# Impact of Pornography Consumption on Sexual Self-Esteem in Tunisian Women

**DOI:** 10.1192/j.eurpsy.2025.2285

**Published:** 2025-08-26

**Authors:** D. Achref, A. Aissa, S. Rouached, R. Jomli

**Affiliations:** 1 Psychiatry department A; 2 Psychiatry department C, Razi Hospital, tunis, Tunisia

## Abstract

**Introduction:**

The consumption of pornographic products is a phenomenon that continues to increase considerably, especially among young people. It is encouraged by easier access to the Internet. On the other hand the concept of sexual self-esteem is a relatively new and little explored subject in the literature. It has emerged across a range of areas such as weight, sexual trauma and physical satisfaction.

**Objectives:**

The aim of our study is to evaluate the impact of pornography consumption on sexual self-esteem in a population of Tunisian women and to determine the associated factors influencing this relationship.

**Methods:**

This is a cross-sectional, descriptive, and analytical study conducted among 107 Tunisian women.

Data were collected using an anonymous self-questionnaire in French, using Google Forms, distributed across various Facebook platforms. It explored sociodemographic data, medical history and sexual health characteristics, sexual behavior, and pornography consumption along with its impact.

For the evaluation of sexual self-esteem, we used the “Sexual Self-Esteem Inventory for Women - Short Form” (SSEI-W-SF) in French language. (Hannier, S et al. Translation and validation study of the French version of the “SSEI-W-SF”. Sexologies. 2022;31.)

**Results:**

The majority of women (76%) are aged between 18 and 30 years, primarily from urban backgrounds (89%), professionally active (81%). In terms of marital status, 43% are single. Most women (60%) have no psychiatric history, 25% are being treated for anxiety disorders, and 20% for mood disorders. More than a quarter (28%) reported experiencing sexual abuse. Regarding sexual education, majority of women (94%) consider its learning essential, although only 11% received structured education at school. Consequently, 80% of women indicated that their main source of sexual education was online. In the studied population, 60% of women reported consuming pornography, primarily in the form of videos (79%). The evaluation of sexual self-esteem using the SSEI-W-SF revealed an average total score of 61.66, ranging from 26 to 82, with a median of 63 and a standard deviation of 10.887. Women with the lowest scores were particularly affected in terms of adaptability and control. We observed a significant correlation between pornography consumption and the absence of sexual education during childhood (p = 0.009). Women who deemed sexual education essential showed lower consumption of pornography (p = 0.027).

Regarding sexual self-esteem, no statistically significant correlation was found between sexual self-esteem and pornography consumption, either for the global sexual self-esteem score (p = 0.809) or for the five spheres of sexual self-esteem assessed separately.

**Conclusions:**

In conclusion, the influence of pornography on sexual self-esteem warrants careful examination, requiring a balanced perspective that addresses both its positive and negative effects on individuals’ well-being.

**Disclosure of Interest:**

None Declared

